# A Simple Knot Technique to Simultaneously Prevent Medial‐Row Suture Loosening and Lateral‐Row Anchor Failure in Double‐Row Rotator Cuff Repair

**DOI:** 10.1002/atn2.70370

**Published:** 2026-10-29

**Authors:** Weidong Wang, Mu Gao, Yingbiao Wang, Sen Li

**Affiliations:** ^1^ Department of Sports Medicine Panyu Hospital of Traditional Chinese Medicine Panyu Hospital of Guangzhou University of Chinese Medicine, Guangzhou Guangdong China

## Abstract

We describe a simple knot‐tying technique to augment fixation during double‐row rotator cuff repair. After standard lateral‐row anchor placement and suture tensioning, the remaining suture limbs are secured with a surgeon's knot positioned flush against the bone‐anchor interface. This knot serves as a mechanical stopper: when the retracted cuff exerts medial traction on the medial‐row sutures, the knot blocks suture slippage at the lateral bone surface, generating a counterforce that neutralizes pullout forces and maintains footprint compression. No additional implants are required. This technique is particularly valuable in osteoporotic bone or massive cuff tears, where lateral‐row anchor fixation is prone to early failure.

## Graphical abstract

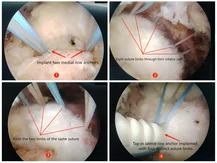

**VIDEO 1:** This video shows a simple knot‐tying technique designed to prevent medial‐row suture loosening and lateral‐row anchor failure during double‐row rotator cuff repair. This technique is particularly applicable and useful in clinical cases involving osteoporotic bone or massive rotator cuff tears, where the risk of suture‐ or anchor‐related complications is relatively higher. Initially, the rotator cuff was repaired using the standard suture‐bridge technique, and the postoperative appearance of the repaired rotator cuff is shown in the accompanying figure. The key steps of our modified technique are as follows: left shoulder arthroscopy was performed in the supine position. After inserting the lateral‐row anchors (STAR, STAR Sports Med, Beijing, China, Tapscrew AK5‐C, 4.5 mm) loaded with sutures from the medial‐row anchors (STAR, STAR Sports Med, FastLock‐φ4.5) into the bone 4 mm beneath the cortical bone, every 2 suture ends were tied with a surgeon's knot (3‐1‐1 configuration). As shown in the postoperative figure, the tied knots were closely apposed to the bone‐anchor interface, serving as a mechanical barrier to prevent suture loosening and anchor failure. Subsequently, we show the verification step: after insertion of the lateral‐row anchors, traction is applied to the sutures that compress the rotator cuff footprint using a suture grasper. This step confirms that the sutures can slip under a specific pulling force, thereby showing failure of both the compression sutures and the lateral‐row anchors. When pulling the sutures with a suture grasper, it is crucial to stabilize the lateral‐row anchors using a knot pusher or the integrated pin/rod of the anchor; this precautionary measure prevents loosening or pullout of the lateral‐row anchors during suture traction. Next, the anchor tail is pressed using the anchor's integral shaft (preattached at manufacture) or a knot pusher, and all sutures are fully tensioned to ensure optimal compression of the rotator cuff footprint. Every 2 suture ends are then tied again using a surgeon's knot (3‐1‐1 configuration). Notably, the knot must sit tightly against the bone‐anchor interface and be embedded in the groove formed by the lateral‐row anchors to maximize its mechanical stability. Throughout the knot‐tying and knot‐pushing processes, forceful pulling along the suture axis should be avoided to prevent loosening of the lateral‐row anchors, thereby ensuring the overall stability of the rotator cuff repair construct. Video content can be viewed at https://doi.org/10.1002/atn2.70370.

Double‐row rotator cuff repair offers superior footprint restoration and greater initial fixation strength compared with single‐row techniques.^1^, ^2^, ^3^, ^4^ However, lateral‐row anchor failure remains a significant clinical concern, especially in osteoporotic humeral heads and osteoporotic bone (*T*‐score < −2.5).^5^, ^6^, ^7^ The cancellous bone of the greater tuberosity provides limited purchase for suture anchor fixation;^8^ when the retracted cuff pulls on the medial‐row sutures, the lateral anchor‐bone interface may fail because of suture slippage or anchor pullout. This ultimately results in the loss of medial‐row tension and failure to maintain footprint compression.^9^ This article shows that rotator cuff repair using suture anchors is the current standard; however, in osteoporotic patients, anchor pullout can compromise surgical outcomes.^10^

To address this biomechanical vulnerability at its source, we describe a simple augmentation technique. By tying the lateral‐row suture limbs in a knot positioned at the bone‐anchor interface, we create a physical barrier that prevents suture slippage and converts destructive pullout forces into compressive forces acting on the anchor itself. This technique requires no additional implants, adds less than 2 minutes to the total operative time, and is compatible with all standard suture anchor systems (Video 1).

## SURGICAL TECHNIQUE

### Step 1: Medial‐Row Anchor Placement

Two medial‐row suture anchors with double sutures (STAR Sports Med, Beijing, China, FastLock‐φ4.5) are inserted into the medial aspect of the rotator cuff footprint (0.5 cm lateral to the articular surface) using standard arthroscopic techniques. The 8 free ends of the 4 sutures are evenly passed through the tendon in a mattress suture pattern, and the free ends of each individual suture are knotted to complete rotator cuff repair at the anatomical neck level (Figure 1, Video 1).

**FIGURE 1:**
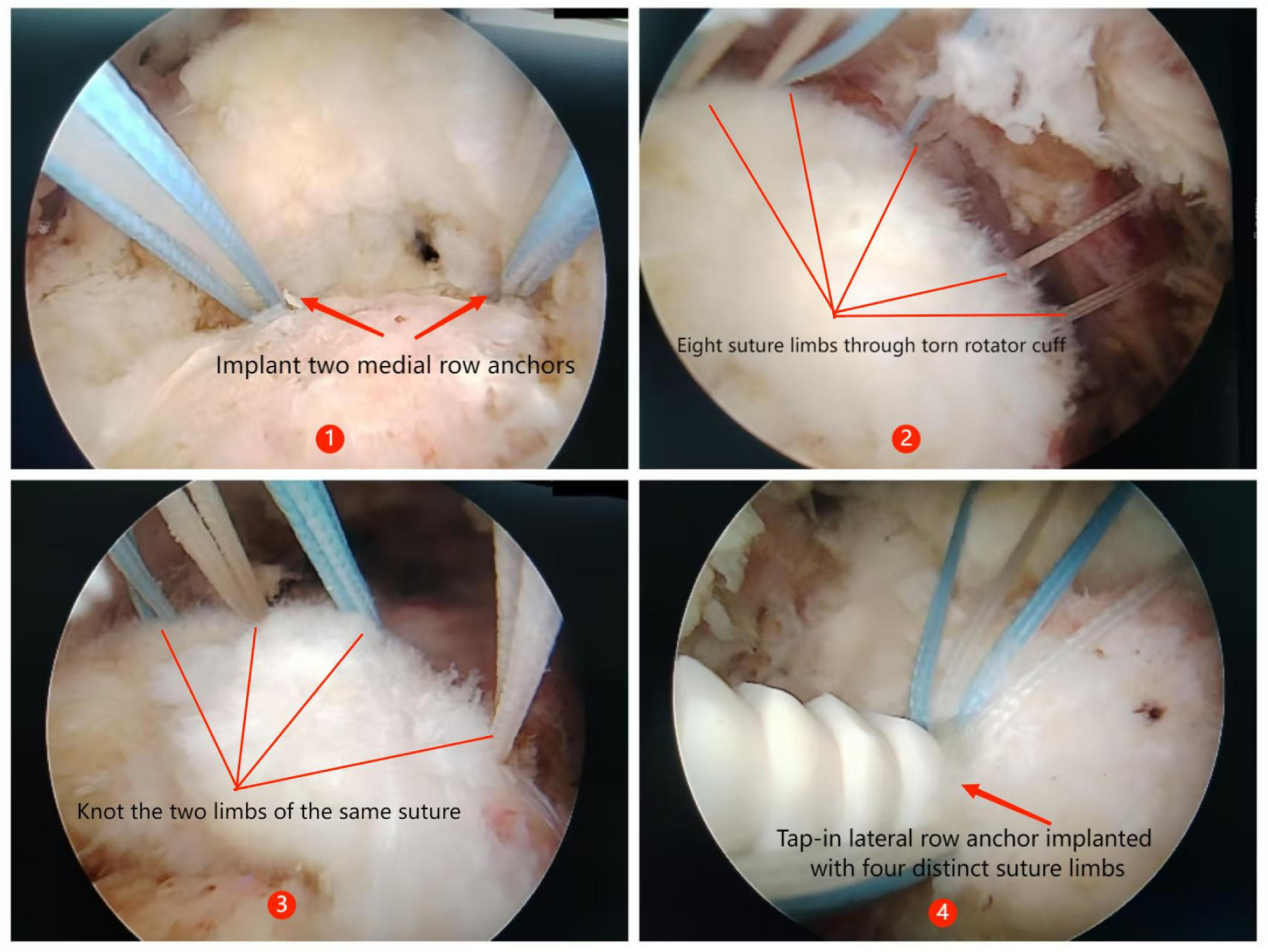
Surgical procedures of medial‐lateral suture‐bridge repair for the right shoulder (supine position, anterolateral viewing portal). (1) Two medial‐row anchor (STAR, FastLock‐φ4.5) implants are placed 5 mm lateral to the anatomical neck. (2) Eight suture limbs are passed through the torn rotator cuff tendon with uniform distribution. (3) The 2 limbs of each single suture are tied to form secure knots. (4) A lateral‐row anchor (STAR, Tapscrew AK5‐C, 4.5 mm) is inserted at the lateral aspect of the greater tuberosity (rotator cuff footprint), loaded with 4 separate suture limbs, to compress the rotator cuff tendon firmly onto the footprint.

### Step 2: Lateral‐Row Anchor Insertion and Suture Tensioning

The free ends of different sutures are divided into 2 groups of 4, retrieved through the lateral portal, and crossed. Two lateral‐row anchors (STAR Sports Med; either Tapscrew AK5‐C, 4.5 mm, or ExtraLock AK7‐D1, 5.5 mm) are placed separately on the lateral ridge of the greater tuberosity with an interanchor distance of >1 cm to achieve initial compression of the rotator cuff footprint (Figures 2 and 3). Importantly, the implanted lateral‐row anchors should be positioned approximately 4 mm below the cortical bone to form a groove (Figure 2(2)); this facilitates embedding of the suture knots into the groove after tying the suture tails (Figure 2(3,4)), thereby preventing knot impingement with the acromion during shoulder abduction.

**FIGURE 2:**
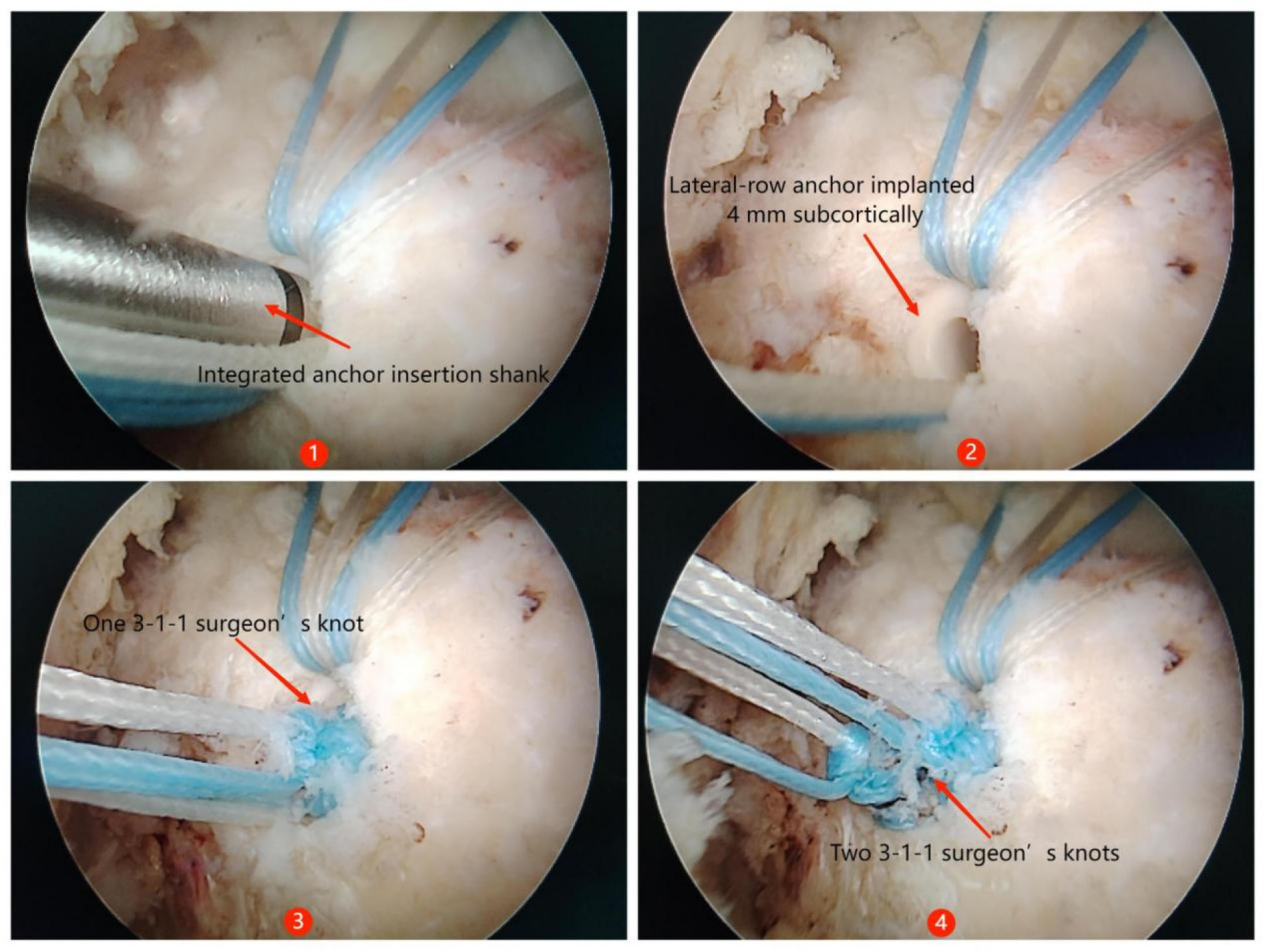
Arthroscopic view through anterolateral portal in supine position: lateral‐row anchor implantation (STAR, Tapscrew AK5‐C, 4.5 mm) for the right shoulder. (1) The dedicated pusher rod of the lateral anchor compresses the anchor body, and all medial‐row sutures are manually tensioned. (2) The pusher rod is separated after all sutures are fully tensioned. (3) The 4 sutures are divided into 2 bundles, and a 3‐1‐1 surgical knot is tied for each bundle. (4) Completion of 3‐1‐1 surgical knots on both suture bundles.

**FIGURE 3:**
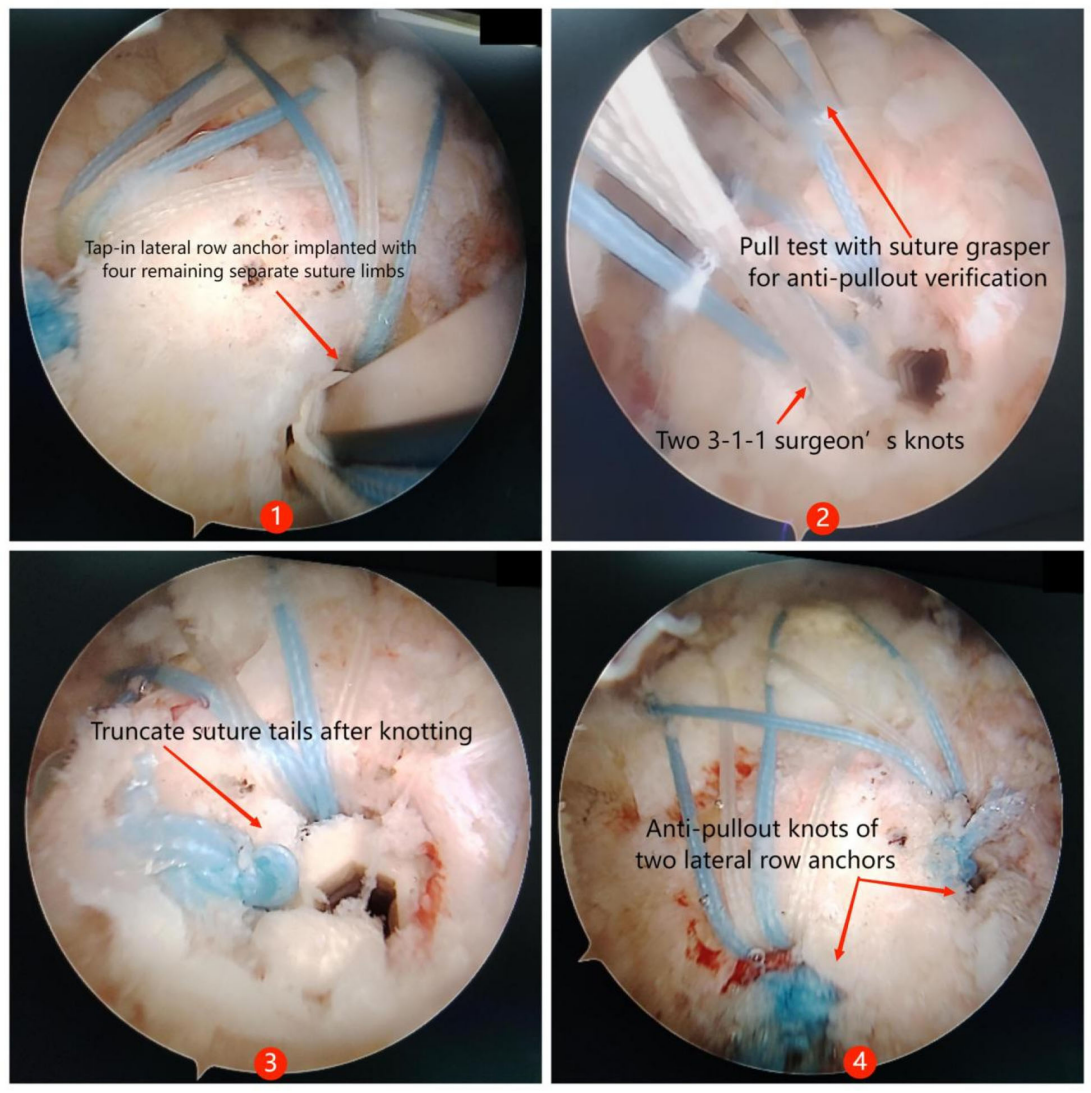
Arthroscopic view through anterolateral portal in supine position: lateral‐row anchor implantation (STAR, ExtraLock AK7‐D1, 5.5 mm) for the right shoulder. (1) The dedicated pusher rod of the lateral anchor compresses the anchor body, and all medial‐row sutures are manually tensioned. (2) A suture grasper pulls the depressed sutures. Traction on sutures induces humeral head motion without loosening of sutures or anchors, confirming the stability of the anchor‐knot construct. (3) Appearance of the anchor‐knot construct after cutting excess suture tails. (4) Completion of 3‐1‐1 surgical knots on both suture bundles. The intact suture bridge fully compresses the rotator cuff onto the tuberosity footprint, with all knots flush to the bone cortex.

### Step 3: Verification of Suture Loosening and Failure Without Knot Tying (This Step Is Optional Intraoperatively)

The suture tails are not transected. A knot pusher or the anchor's integral shaft (preattached at manufacture) is used to stabilize the anchor tail, while a suture grasper is employed to pull the sutures, compressing the rotator cuff footprint. This step verifies that sutures loosen under a certain degree of pulling force, leading to failure of rotator cuff footprint compression (Figures 4 and 5, Video 1).

**FIGURE 4:**
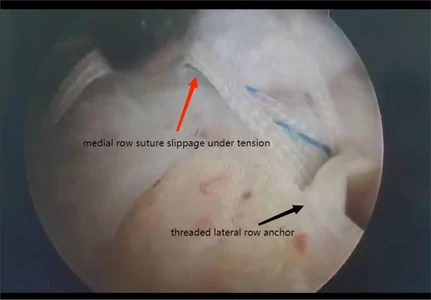
Arthroscopic view of the right shoulder through the posterolateral portal reveals medial‐row suture slippage under tensile load and a threaded lateral‐row anchor (STAR, ExtraLock AK7‐D1, 4.75 mm). The red arrow indicates the medial‐row suture slippage under tension, and the black arrow marks the threaded lateral‐row anchor in the arthroscopic field of view.

**FIGURE 5:**
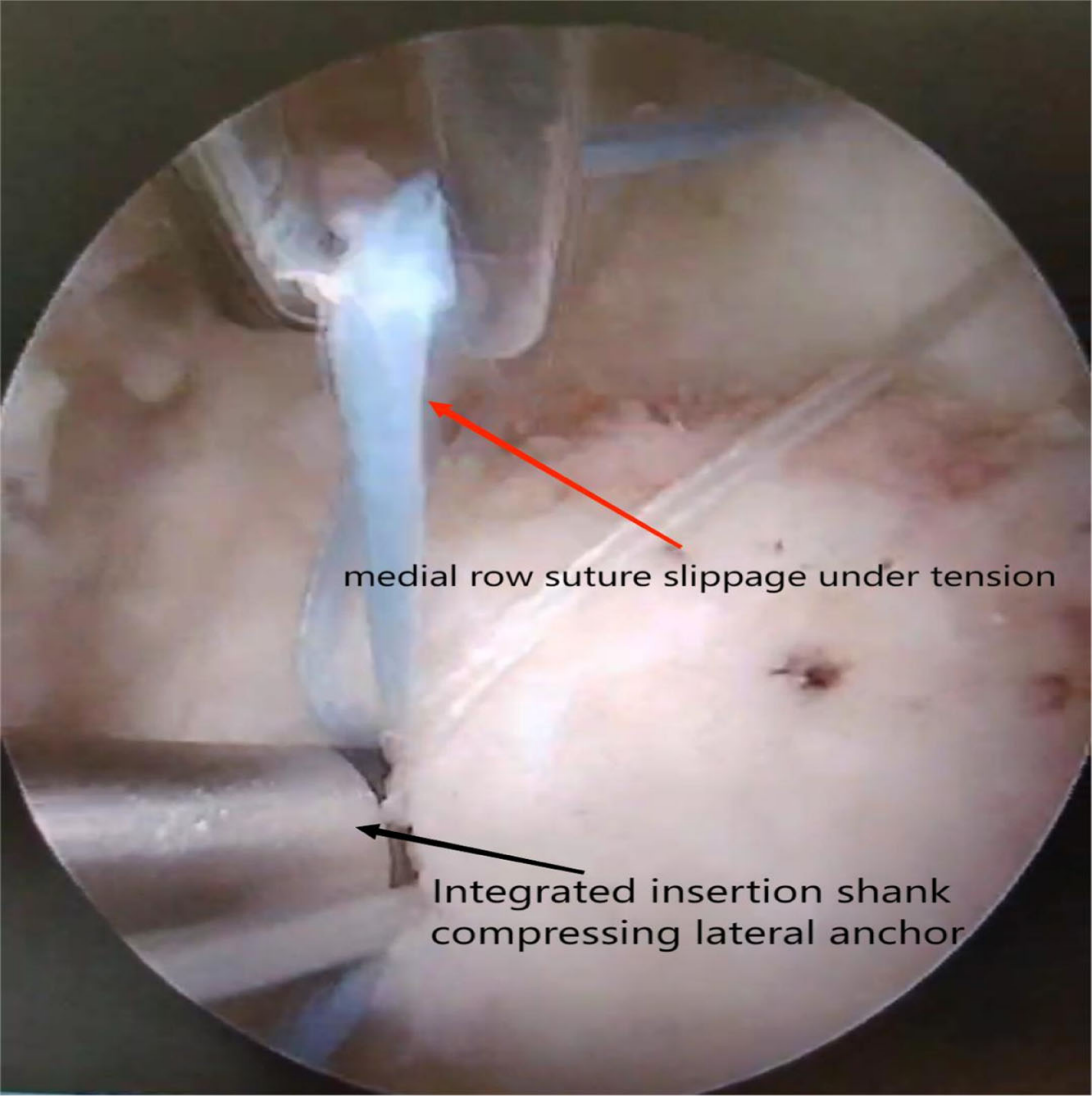
Arthroscopic observation of the right shoulder through the anterolateral portal in the supine position for traction testing of tensioned medial‐row sutures. After the dedicated pusher rod of the lateral anchor (STAR, Tapscrew AK5‐C, 4.5 mm) compresses the lateral anchor body and all sutures are tensioned, traction applied to taut sutures with a suture grasper results in easy suture loosening. This finding shows insufficient pullout resistance at the anchor‐bone interface of the lateral anchor, predisposing the entire repair construct to failure.

### Step 4: Knot Tying at Bone‐Anchor Interface (Key Step)

The anchor tail is stabilized with a knot pusher or the anchor's integral shaft (preattached at manufacture), all sutures are fully tensioned, and every 2 suture ends are tied using a surgeon's knot (3‐1‐1 configuration) (Figure 6). The knot is positioned tightly against the bone‐anchor interface and embedded in the groove formed by the lateral‐row anchors (Figure 7(1)). During knot tying and knot pushing, forceful traction on the suture axis is avoided to prevent loosening of the lateral‐row anchors (Video 1).

**FIGURE 6:**
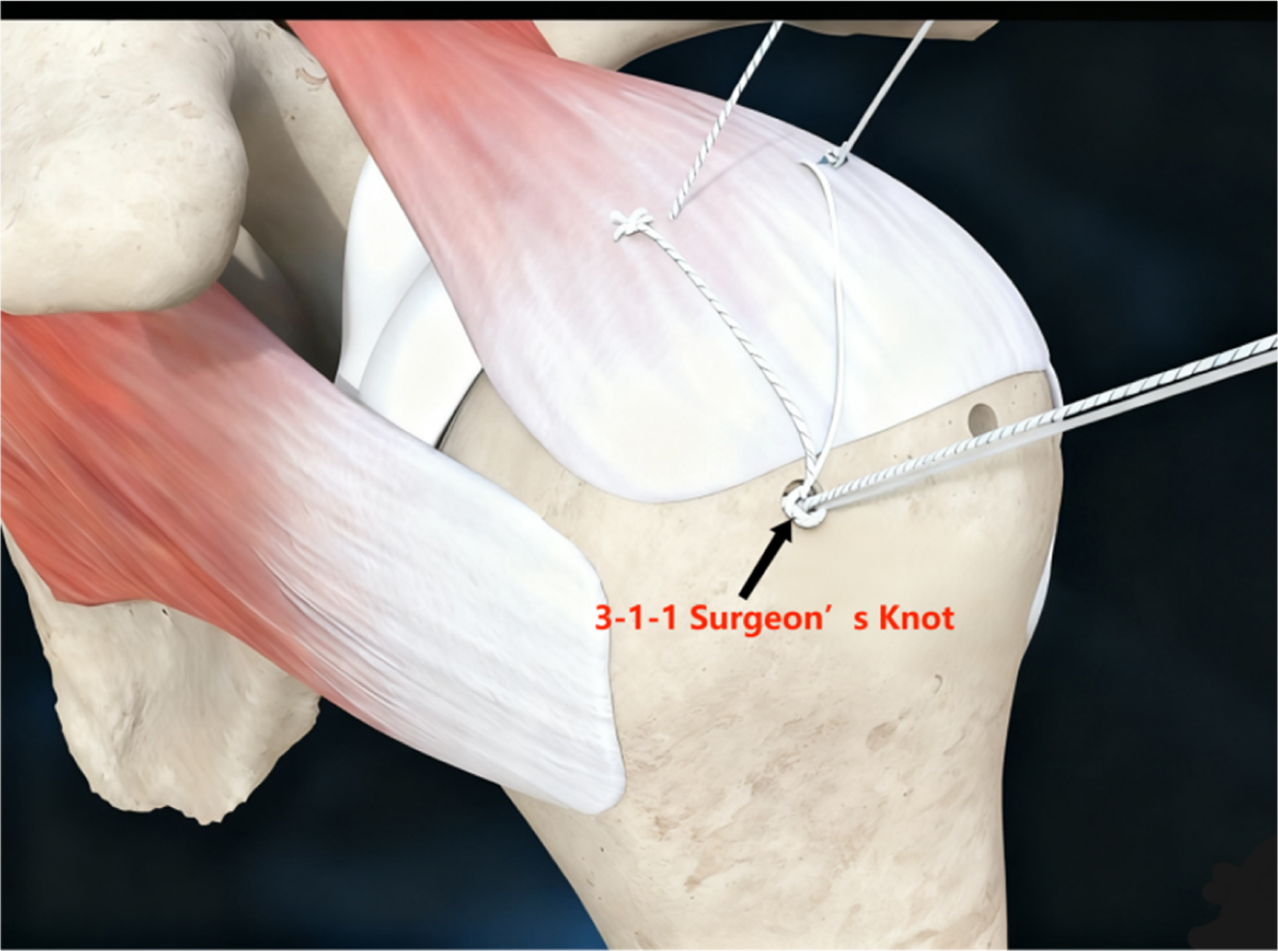
The 3‐1‐1 surgeon's knot configuration (left shoulder model). The knot consists of 3 initial throws followed by 2 additional half hitches (1‐1), providing secure knot fixation in rotator cuff repair. Step 1: 3‐throw locking base: 3 consecutive throws of the long suture limb around the short limb, tightened to form a strong initial locking structure. This is the key feature that distinguishes the 3‐1‐1 knot from the conventional 2‐1‐1 surgeon's knot. Step 2: first half hitch. One standard reverse half hitch, appropriately tightened to prevent loosening of the locking knot. Step 3: second half hitch (final locking). An additional reverse half hitch, fully tightened to complete the 3‐1‐1 configuration.

**FIGURE 7:**
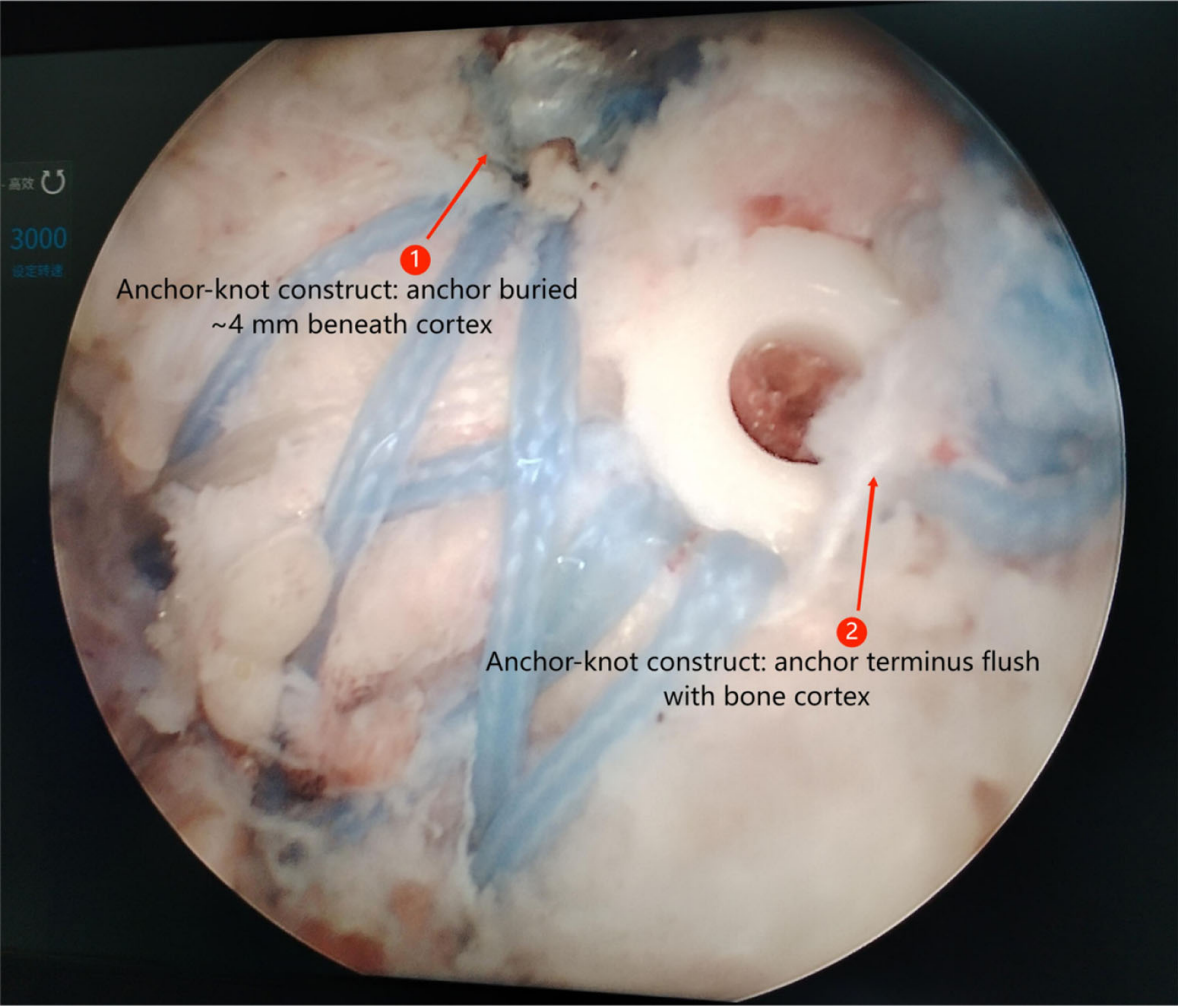
(1) Recessed insertion of lateral‐row anchor and suture knotting under left shoulder arthroscopy. Anchor insertion: the lateral‐row anchor (STAR, Tapscrew AK5‐C, 4.5 mm) with sutures from the medial‐row anchor (STAR, FastLock‐φ4.5) was inserted into the target bone. The insertion depth was precisely controlled to bury the anchor body approximately 4 mm beneath the cortical bone, ensuring secure engagement between the anchor and bone to prevent loosening. Suture grouping: the suture tails of the anchor were grouped and secured using a 3‐1‐1 configuration surgeon's knot, guaranteeing tight and evenly stressed knots, maintaining appropriate suture tension, and achieving reliable fixation between the tissue and bone. (2) Flush insertion of lateral‐row anchor and suture knotting. Anchor insertion: the lateral‐row anchor (STAR, Tapscrew AK5‐C, 4.5 mm) with sutures from the medial‐row anchor (STAR, FastLock‐φ4.5) was inserted into the bone. The anchor position was adjusted so that the distal end of the anchor was flush with the cortical bone surface, ensuring uniform insertion depth and stable fixation. Suture grouping: the suture tails of the anchor were grouped to clarify the function and orientation of each suture bundle. Knotting: a standardized 3‐1‐1 configuration surgeon's knot was used to complete the knotting, with appropriate suture tension to ensure satisfactory tissue reduction and secure fixation. Advantages of (1): reduces knot impingement. Disadvantages of (1): prone to cortical bone cutting by the medial‐row sutures. Advantages of (2): less prone to cortical bone cutting by the medial‐row sutures. Disadvantages of (2): relatively higher risk of knot impingement.

### Step 5: Verification of Mechanical Block

After arthroscopic suture repair as shown in Figure 4, the medial‐row sutures are pulled with a suture grasper until humeral head mobility is observed. At this point, no lateral‐row anchor loosening, knot displacement, or loss of medial‐row suture tension was noted, confirming that the knots are sufficient to resist the applied pulling force (Video 1).

## DISCUSSION

This technique addresses a key biomechanical weakness in double‐row rotator cuff repair.^4^ Conventional lateral‐row fixation relies solely on frictional engagement between anchors and cancellous bone, a simplistic mode with poor overload resistance. Once microloosening or stripping occurs at the anchor‐bone interface, medial‐row tension is lost, footprint compression fails, tendon gaps develop, and repair breakdown ensues.^9^ Although lateral‐row anchors with internal locking bolts for suture locking are commercially available, they remain prone to loosening under load when implanted in osteoporotic bone. Our modified knot constructs an active mechanical stopper on the lateral bone surface, converting axial pullout force into intraosseous compressive force acting on the anchor, optimizing the load‐bearing pattern (Figure 7(1,2)). The principle is as follows: retracted rotator cuff tendons exert persistent medial traction on sutures; the knot exceeds the anchor‐bone gap and cannot slide inward. The resultant counterforce seats the anchor deeper into cancellous bone, counteracting pullout and stabilizing the construct. Thus, sustained medial‐row tension and continuous footprint apposition are maintained, providing a stable environment for tendon healing. This study indicates that knotted suture‐bridge repair provides superior early biomechanical performance.^11^

Compared with traditional double‐row fixation, this technique has multiple clinical and biomechanical merits and wider applicability (Table 1). First, it enables fault‐tolerant fixation, eliminating the “series failure” flaw of conventional constructs. Traditional fixation fails entirely once the lateral interface loosens, whereas our stopper design retains medial tension and footprint compression under partial loosening, greatly raising the failure threshold. Second, it addresses osteoporotic fixation difficulties. For osteoporotic bone (*T*‐score < −2.5) with poor bone mass and weak anchor purchase, standard friction‐based fixation easily loosens and migrates. Via force transformation, our design deepens anchor impaction and stabilizes fixation in osteoporotic bone beds.^12^ Third, it fits high‐load, severely retracted lesions. For large rotator cuff tears over 3 cm under sustained tension, this technique offsets chronic traction, lowering micromotion and retear risk.^13^ Recent evidence further confirms that high repair tension and advanced age are independent risk factors for poor healing after rotator cuff repair, underscoring the importance of tension‐neutralizing strategies in massive tear fixation.^14^ It also works for revision cases with deficient bone stock, boosting fixation without extra augmentation implants. Fourth, it is minimally invasive and economical. No additional implants are needed intraoperatively, avoiding foreign‐body reactions, implant shift, and secondary surgery while easing patient costs. Fifth, it is time‐efficient and broadly applicable. Extra knot tying takes under 2 minutes without extending surgery or anesthesia time. Compatible with all standard suture anchors and requiring no specialized tools, it has a gentle learning curve for widespread clinical use.

**TABLE 1:** Advantages and Disadvantages

**Advantages**	**Disadvantages**
Resolves the key biomechanical defect of conventional double‐row rotator cuff repair by converting axial pullout force into intraosseous compressive force.	Theoretical risk of subacromial impingement caused by prominent knot configuration.
Provides fault‐tolerant fixation and avoids serial structural failure despite partial lateral interface loosening.	Overtightened sutures may cut through osteoporotic bone, resulting in secondary anchor instability.
Improves fixation stability in osteoporotic bone (*T*‐ score < −2.5) with insufficient bone stock.	Strictly dependent on an intact lateral humeral cortical ridge; not applicable for comminuted ridge fractures.
Reinforces fixation for large retracted tears (>3 cm) and challenging revision cases without additional implants.	Medium‐ and long‐term clinical outcomes and structural durability remain unvalidated.
Requires no augmented implants, avoids foreign‐body reaction, and reduces patient economic burden.	Precise knot tension control is required, with an inherent technical learning curve.
Adds less than 2 min of operative time and is universally compatible with all standard anchor systems.	Improper tension modulation may offset the biomechanical advantages of the modified technique.

min, minutes.

Nevertheless, this technique has clear technical risks, limitations, and contraindications requiring strict patient selection and careful operation. First, bulky knots may cause subacromial impingement. Because knots act as mechanical stoppers, ill‐formed protruding knots narrow the subacromial space, inducing shoulder friction, pain, and limited motion. No related complications occurred in our cohort, yet this risk still exists theoretically. Second, overtightened sutures can cut through osteoporotic bone. Brittle osteoporotic cortex bears high shear stress under strong tension, easily triggering suture penetration, bone defects, anchor loosening, and repair failure.^4^ Third, it has strict anatomical restrictions. Relying on intact lateral tuberosity cortical bone for support, the technique only applies to patients with intact bone beds and is contraindicated for comminuted greater tuberosity fractures with damaged lateral ridges, which prevent valid stopper construction. Fourth, clinical evidence is inadequate. Only short‐term clinical and biomechanical data are available; mid‐ to long‐term follow‐up is missing to confirm long‐term construct stability, late retear rates, and soft‐tissue tolerance, needing further research. Fifth, a learning curve exists. Proper control of knot tension and anchor depth governs surgical results, and improper tension will eliminate its benefits.

To maximize benefits and mitigate complications, standardized operative protocols must be rigorously followed (Table 2). Lateral anchors are routinely inserted 4 mm beneath the cortex for flush knot placement, reducing impingement risk (Figure 7(1)). For selected cases where medial‐row sutures tend to cut through the cortex during tensioning, anchors may be placed at the cortical plane, yet meticulous knot contouring is mandatory to minimize protrusion (Figure 7(2)). A stable 3‐1‐1 surgeon's knot is recommended instead of sliding knots. After final medial‐row tensioning, a hook probe must confirm the structural integrity of the mechanical stopper without sliding gaps to rule out occult failure. Adequate suture limb length and cross‐suture configuration ensure uniform load distribution across the footprint. Deviation from these protocols may result in occult fixation failure, early repair loosening, and heterogeneous footprint compression, adversely affecting healing quality.^5^ Preoperative assessment of tear characteristics and patient‐specific risk factors, including Goutallier grade and coracohumeral distance, remains essential for appropriate patient selection and optimizing outcomes.^15^ Future quantitative biomechanical studies are warranted to precisely quantify the augmentative effect of this technique, and long‐term follow‐up is needed to refine clinical indications and standardized operative criteria.

**TABLE 2:** Pearls and Pitfalls

**Pearls**	**Pitfalls**
Position the knot flush with the cortical surface, not above it.	Knot placed too proud → increased impingement risk.
Use a surgeon's knot (3‐1‐1) for security; avoid sliding knots.	Sliding knots may loosen under cyclic loading.
Tighten the knot to create a slight indentation in the bone without cutting through it.	Overtightening → suture cuts through the bone.
Verify the mechanical block with a probe before final medial‐row tying.	Failure to verify → unrecognized fixation failure.
Leave adequate suture length for knot tying (≥3 mm for each limb).	Short suture limbs → insecure knots and early failure.
Cross the sutures before tensioning for even load distribution.	Parallel sutures → uneven footprint compression.

## DECLARATION OF GENERATIVE AI AND AI‐ASSISTED TECHNOLOGIES IN THE WRITING PROCESS

During the preparation of this work, the authors used Doubao (general version) to (1) translate the original manuscript into English and (2) assist in organizing tables. After using this tool/service, the authors reviewed and edited the content as needed and take full responsibility for the content of the publication.

## DISCLOSURES

The authors (W.W., M.G., Y.W., S.L.) declare that they have no known competing financial interests or personal relationships that could have appeared to influence the work reported in this article.
